# Using GFP Video to Track 3D Movement and Conditional Gene Expression in Free-Moving Flies

**DOI:** 10.1371/journal.pone.0040506

**Published:** 2012-07-19

**Authors:** Reza Ardekani, Yichuan Michelle Huang, Prathamesh Sancheti, Ramunas Stanciauskas, Simon Tavaré, John Tower

**Affiliations:** Molecular and Computational Biology Program, University of Southern California, Los Angeles, California, United States of America; Imperial College London, United Kingdom

## Abstract

**Background:**

In vivo imaging and quantification of fluorescent reporter molecules is increasingly useful in biomedical research. For example, tracking animal movement in 3D with simultaneous quantification of fluorescent transgenic reporters allows for correlations between behavior, aging and gene expression. However implementation has been hindered in the past by the complexity of operating the systems.

**Results:**

We report significant technical improvements and user-friendly software (called FluoreScore) that enables tracking of 3D movement and the dynamics of gene expression in adult Drosophila, using two cameras and recorded GFP videos. Expression of a transgenic construct encoding eGFP was induced in free-moving adult flies using the Gene-Switch system and RU486 drug feeding. The time course of induction of eGFP expression was readily quantified from internal tissues including central nervous tissue.

**Conclusions:**

FluoreScore should facilitate a variety of future studies involving quantification of movement behaviors and fluorescent molecules in free-moving animals.

## Introduction

In vivo monitoring of fluorescent and luminescent reporter molecules is proving increasingly useful in the study of cell behavior and physiology, including stem cell dynamics, cancer, neurobiology and aging [1]–[4]. For example, transgenic constructs that drive expression of fluorescent molecules such as GFP allow for non-invasive and longitudinal assays of gene expression [5]. We have previously reported methods that allow for 3D video tracking of *Drosophila melanogaster* movement behaviors [6], [7], while simultaneously quantifying gene expression using fluorescent reporter proteins [8], [9]. Here we report technical and software improvements that enable increased sensitivity with a two-camera configuration and a user-friendly interface. In addition to tracking 3D movement, these methods allowed for quantification of GFP reporter induction in internal tissues in response to drug feeding and transcriptional activation in free-moving flies.

## Results

### Design and Implementation

**Figure 1:**
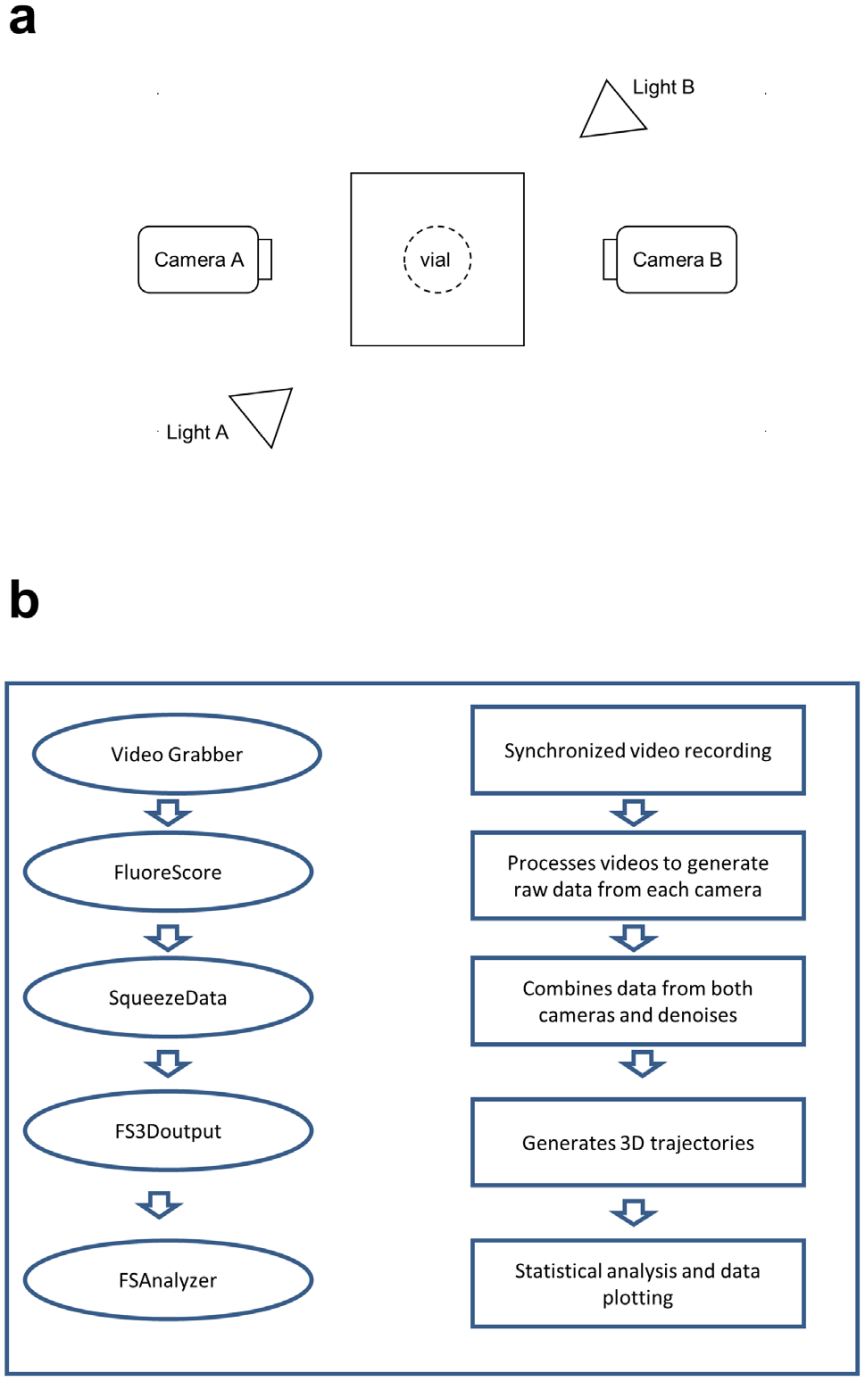
Overview of system and data analysis. (a) Diagram of tracking hardware arrangement. (b) Outline of data acquisition and analysis.

The hardware set-up consists of two blue LED lights (Luxeon V Star) and two video cameras (Grasshopper type GRAS-20S4C, Point Grey Research, Scottsdale, AZ), connected with FW800 PCI-E cards to an HP desktop computer with two Intel 64-bit Quad-Core Xeon Processors (2.13 GHz/Core) and 6GB of RAM that runs Windows 7 Enterprise (64bit). The two cameras are directed at an observation chamber consisting of a standard glass Drosophila culture vial. The two LED lights are directed at the chamber, offset from the cameras by 30 degrees (Figure 1a). For detection of GFP the MDF-GFP filter set is used (Thorlabs Inc, Newton, NJ). The MF469-35 filters are fixed in front of the LEDs to limit the light illuminating the flies to a range of approximately 452 nm to 486 nm, which overlaps the eGFP absorption peak at approximately 488 nm. Each camera has an Edmund optics 8 mm Megapixel fixed focal lens. The MF525-39 filters are fixed in front of the lenses to limit the light being recorded as video to the range of approximately 510 nm to 548 nm, which overlaps the emission peak for eGFP at approximately 509 nm. Using these filters, reflected light should be eliminated, and video should consist only of fluorescent light emitted from the flies. The system is also designed to allow for analysis of red and blue fluorescent reporter proteins, by using alternative light and filter sets. The lights, cameras and observation chamber were placed in darkness by covering with a cardboard box, with one side that opens and closes to allow access to the chamber.

The FluoreScore software suite is divided into several modules to facilitate use and future upgrades (Figure 1b). Video is captured using FlycapSDK software, which is provided with the cameras by Point Grey Research. The cameras are calibrated using Camera Calibration Tools software, freely available at http://www.cs.ucl.ac.uk/staff/Dan.Stoyanov/calib/(D. Stoyanov, personal communication). In addition, we provide five custom software modules, written in Visual Studio C++ (2005). VideoGrabber controls FlycapSDK to allow synchronous initiation and recording of video (http://code.google.com/p/video-grabber/). CalibHelp converts the output from Camera Calibration Tools to a projection matrix that can be used by our software. FluoreScoreSQ further processes the data by removing objects smaller than a user-chosen threshold size, and combines data from the two camera views. FluoreScore3D generates 3D tracking using the 2D values from the two cameras. FloureScore analyzes the videos and produces outputs files in.csv format. The FluoreScore output includes pixel number and intensity values for the detected fly or group of flies, as well as position values (X, Y) when tracking movement of single flies. Finally, FsAnalyzer is an R script that performs basic statistical analyses and calculates motion parameters including velocity and frequency of directional heading changes, and plots the data. Additional details on hardware and software and detailed step-by-step instructions are provided in supplementary materials, experimental protocol (Protocol S1).

**Figure 2:**
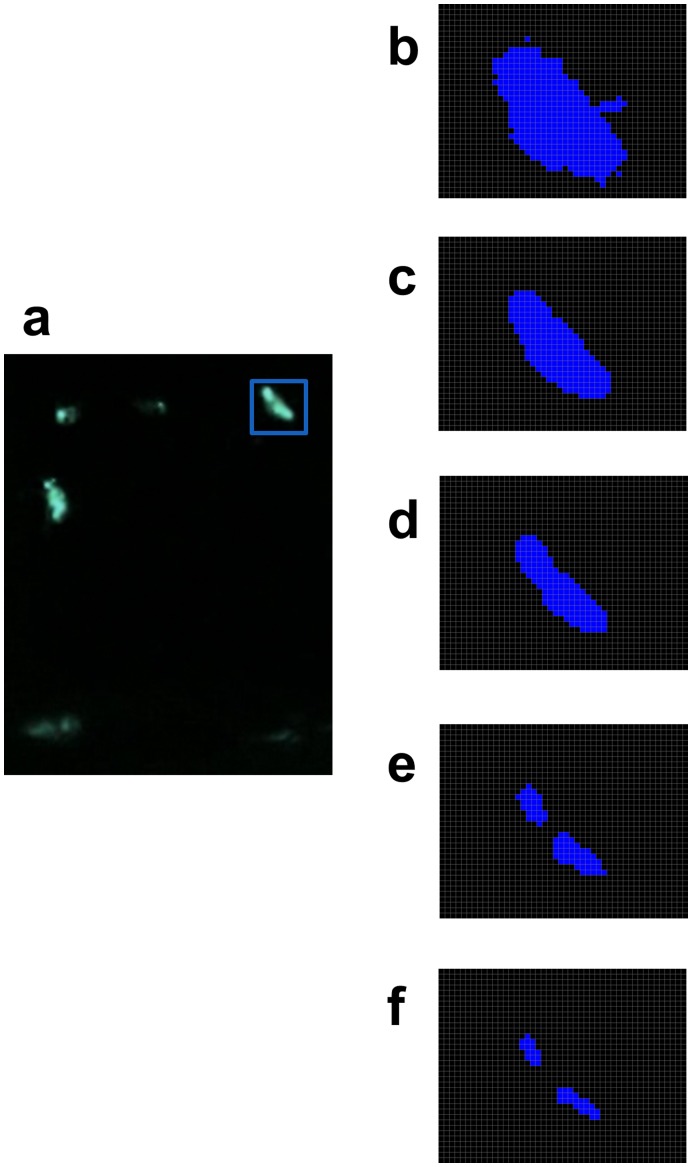
Identifying fluorescent animals and quantifying fluorescence in recorded videos. (a) A single frame of video is presented from tracking six flies with tissue-general eGFP expression (additional details of experiment presented in Figure 6a). (b-f) Silhouette detected for a single fly area (marked by blue box in a) with different values of threshold (b) 5, (c) 50, (d) 100, (e) 150, (f) 175.

The FluoreScore software analyzes recorded videos in several steps; the first step is to detect the flies in the videos. In each of the two videos, the flies are detected based upon their appearance as a bright silhouette against a dark background and movement between frames (Figure 2). A running Gaussian average approach is employed, and the background is modeled independently at each pixel; this model fits a Gaussian distribution to the intensity values of the pixel in previous frames. A selective background update approach is used, which provides a balance between stability to changes in background and speed of updating the background. Once a background model is obtained, a binary change mask is calculated by subtracting background from the current frame and applying a threshold to identify the foreground pixels. Finally silhouettes of flies are identified by detecting groups of adjacent foreground pixels in the binary change mask (Figure 2). In practice there are often stray background pixels in the binary change mask. To remove them, an erosion and dilation morphology operation is performed on the change mask using a 3×3 pixel square structural element [10]. This step removes small components (e.g. single pixels) from the change mask and leaves larger components (i.e., detected flies) essentially unchanged. Moreover, to have a better model of background for the first frame of video 1% of the total frames are sampled at even intervals from the video and used to calculate a background model. This model is used as the initial background and is updated as described above. The threshold value should be set such that flies are detected as foreground without detecting background pixels, and the value of the threshold can be different under different lighting conditions. To facilitate determination of the optimal threshold, the FluoreScore GUI includes a sliding bar that allows the user to see what will be detected with different values of the threshold (Figure 2). Once the optimal threshold is chosen for a particular setup and lighting conditions it should be kept the same across multiple videos in a given experiment to make the data comparable.

After detecting the flies, intensity values (between 0 and 256) are calculated for red, blue and green channels for pixels that represent flies (Figure 2). For each channel the pixels are separated into four bins based on the pixel intensity values. For example, for the green channel, pixels are separated into bins G1, G2, G3 and G4 of increasing intensity. For each fly, the histogram of pixel intensity values is calculated along with the sum of intensities for each bin of the histogram. The reason a simple mean is not used for summarizing intensity information is that for some experiments fluorescence is present in differently sized regions in different flies, and using a simple mean would degrade the signal. For most experiments, the difference between flies with fluorescence and flies without fluorescence is the pixels that fall in the bins with higher intensity (G3 or G4), whereas background pixels will fall into the lower intensity bins (G1 or G2). Therefore, in practice, to quantify the relative fluorescence of two or more samples, the highest bin that has pixels for all samples is used (e.g., G4). If that bin does not contain pixels for each sample, the values from the next lowest bin are added (e.g. G4 plus G3). Finally, the size (in pixels) of each detected component (i.e., fly) is calculated, and the 2D center of mass (centroid) is determined to use for positional tracking. Once these raw data are acquired, the FluoreScoreSQ program eliminates detected components that are smaller in size than a user-chosen threshold (in pixels), and combines the data from the two camera views.

**Figure 3:**
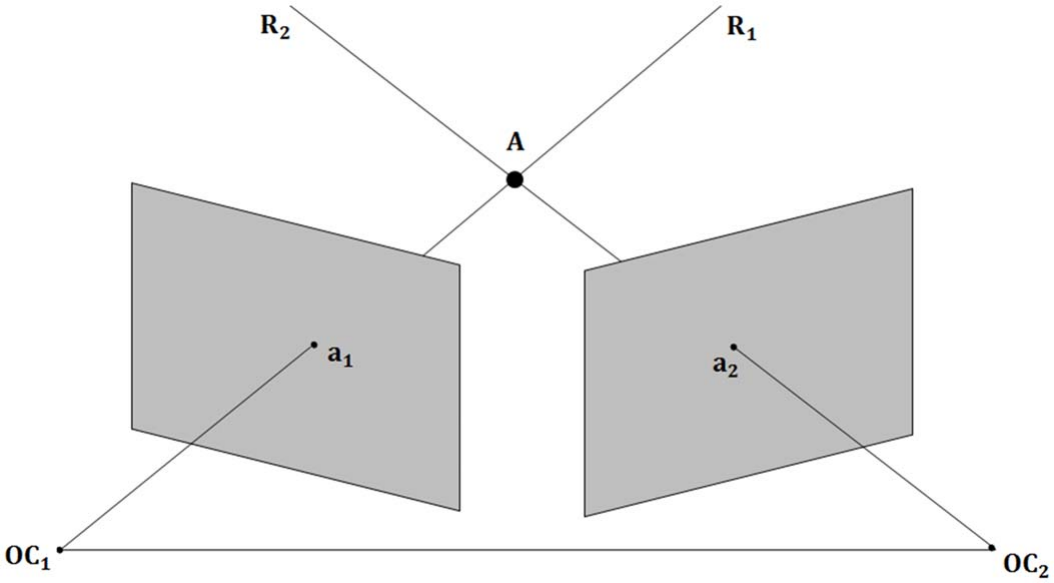
Diagram of 3D reconstruction using two cameras. 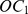
and 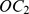
represent the optical centers of Cameras 1 and 2, respectively. 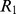 and 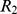
are the 3D rays that pass through optical centers 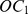
and 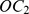, and their projection onto image planes are 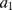
and 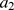, respectively. 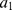
is the 2D position of a fly in view one and 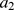 is the 2D position of the fly in view 2. 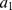
and 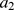
are obtained by FluoreScore. Finally, 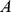 is the actual 3D position of the fly that is the intersection of 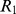
and 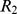.

Depending upon the position and body orientation of the fly, there are sometimes frames in which the fly is not visible in one of the cameras. However, for frames where there are 2D measurements from both views (the centroid for the detected fly), the 3D position of the fly is calculated by the FS3Doutput software (Figure 3). This allows tracking of 3D movement patterns as well as calculation of movement parameters, including velocity and frequency of directional heading changes (alpha), using FsAnalyzer.

### 3D Tracking using GFP Reporter Expression in Retina

**Figure 4:**
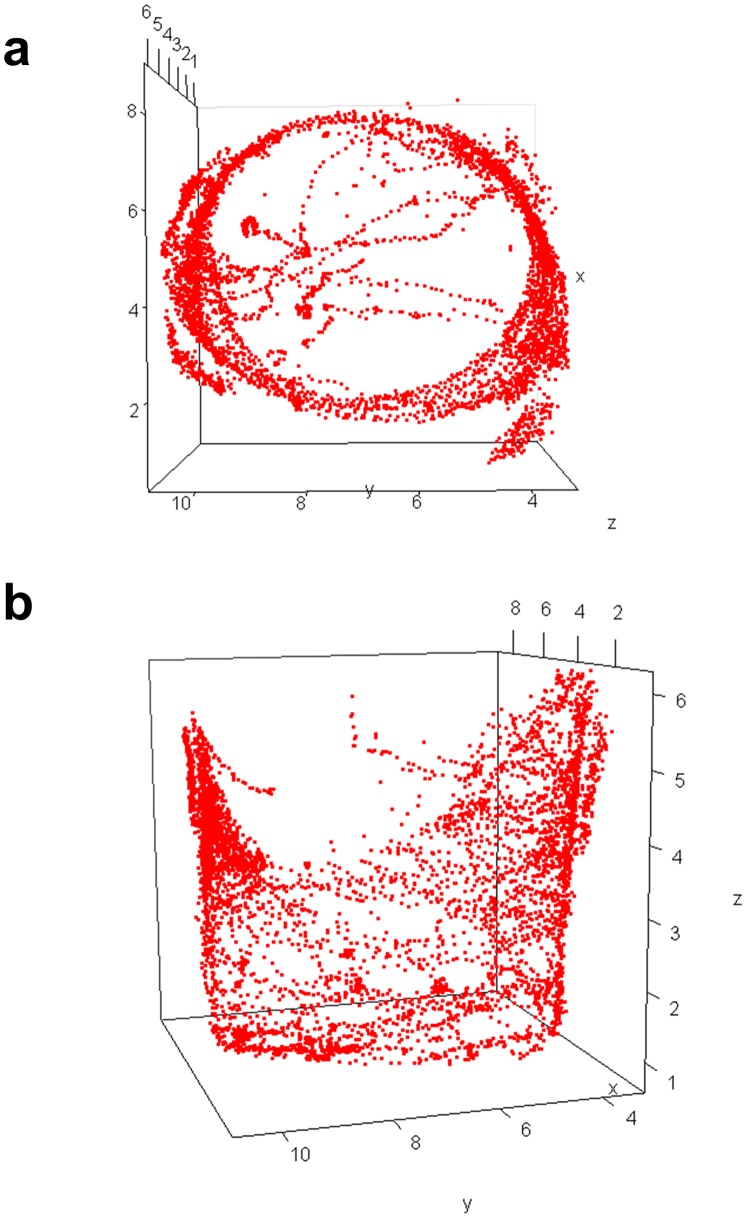
Tracking 3D fly movement. A single 3×P3-GFP male fly was tracked for 5 minutes using GFP fluorescence and the data was analyzed using FluoreScore and FluoreScore3D. The position of the fly is plotted (Red squares). Points represent the 3D position of the fly in each frame of video for which 2D information was available from both cameras. (a) Top view. (b) Side view.

To demonstrate 3D tracking using GFP fluorescence the 3×P3-eGFP transgenic reporter line was used [11]. In this line a synthetic promoter containing three binding sites for the Eyeless/PAX6 transcription factor drives expression of eGFP specifically in retinal tissue. Video of a single male fly was recorded for 5 minutes and the data were analyzed using FluoreScore and FluoreScore3D. eGFP fluorescence was readily quantified and yielded pixel intensities in the G4 bin. FS3D output and FsAnalyzer yielded a plot of fly movement throughout the cylindrical space of the vial (Figure 4). *Drosophila melanogaster* tend preferentially to explore the outer boundaries of their containers, a behavior known as “centrophobism” [12]. Notably the shape of the plotted trajectories corresponds approximately to the contours of the cylindrical observation vial, indicating relatively accurate tracking.

### Tracking Transcriptional Induction in Internal Tissues

**Figure 5:**
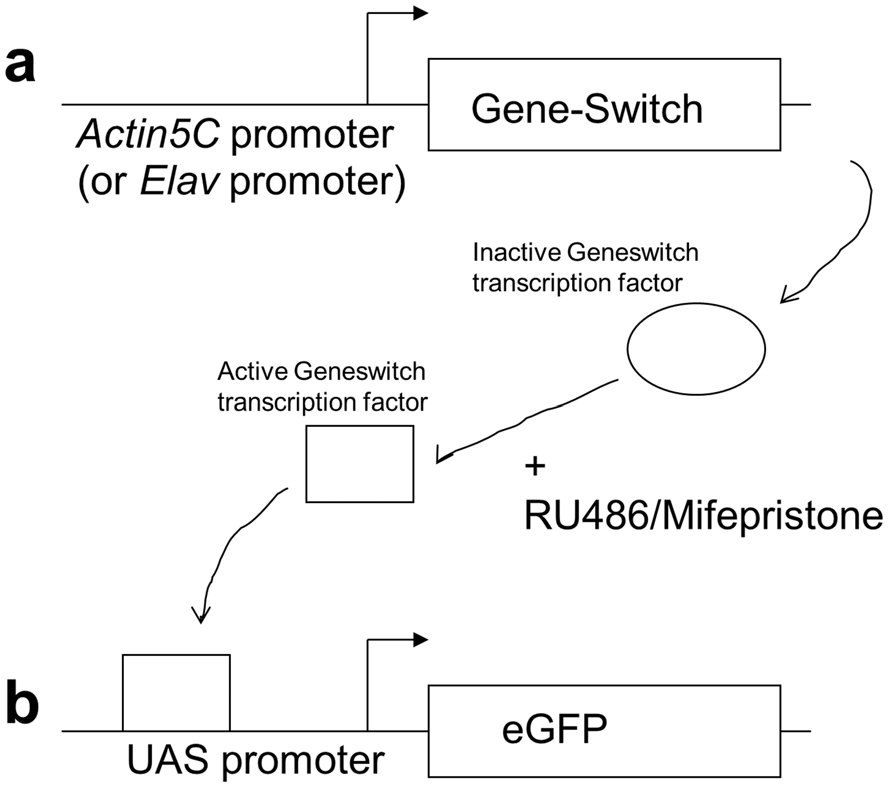
Diagram of Gene-Switch conditional gene expression system. The transgenic flies contained two constructs: a “driver” construct (either Actin-GS[255B] or Elav-GS) (a), and the UAS-eGFP “reporter” construct (b). The Actin-GS[255B] construct drives expression of the Gene-Switch transcription factor in all the somatic tissues of the fly, whereas the Elav-GS construct drives expression of Gene-Switch specifically in neurons. The Gene-Switch transcription factor is inactive until it comes in contact with the drug RU486, which is administered to the flies by feeding. Once activated by RU486, the Gene-Switch factor binds to the UAS sites in the promoter of the reporter construct, activates transcription, and drives high-level expression of the fluorescent protein eGFP.

**Figure 6:**
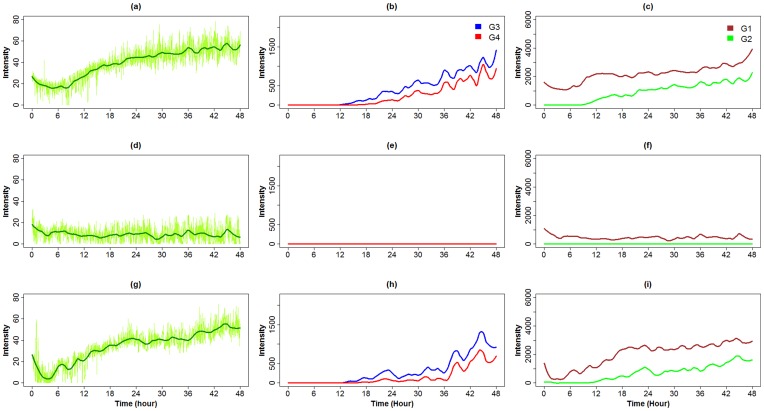
Tracking transgenic reporter induction in response to drug feeding. Groups of six flies were tracked for 48 hours and the data were collected as 48 one-hour videos from each camera. The videos were analyzed using FluoreScore and the average GFP intensity per minute is plotted in light green (a, d, g). In addition data was smoothened using LOESS (with span  = 0.1 and degree  = 2.0) and is indicated with solid lines (a-i). (a-f) FluoreScore assay of UAS-eGFP reporter flies using the tissue-general Actin-GS[255B] driver. A group of six male flies was assayed for 48 hours in the presence of drug (a-c), and a control group of six male flies was assayed in the absence of drug (d-f), and the time course of eGFP induction was quantified. (g-i) FluoreScore assay of UAS-eGFP reporter flies using the neuron-specific Elav-GS driver. A group of six male flies was assayed for 48 hours in the presence of drug and the time course of eGFP induction was quantified.

Experiments were undertaken to determine if FluoreScore could quantify the time course of GFP expression when transcription of a gene was activated in vivo. The Gene-Switch conditional transgenic system [13]–[16] was used to cause conditional expression of a reporter transgene encoding GFP (diagrammed in Figure 5). In the first experimental design the driver line Actin-GS[255B] was used [16], [17]. Here the powerful *Actin5C* gene promoter drives expression of the Gene-Switch transcription factor in all somatic tissues of the adult fly. Gene-Switch is a chimeric transcription factor consisting of a mutated human progesterone receptor-ligand binding domain, the yeast GAL4 DNA binding domain, and part of the transcriptional activation domain of the human p65 protein [18]. The Gene-Switch transcription factor is inactive until it comes in contact with the drug RU486/Mifepristone, whereupon it undergoes a conformation change and can bind to sequence-specific UAS sites engineered into the fly genome. The flies also contained a second transgene where a UAS-containing promoter drives expression of enhanced-fluorescence GFP (eGFP) [19]. In this way feeding the flies the drug RU486 causes the Gene-Switch transcription factor to activate transcription of the UAS-eGFP transgene. Before beginning the experiment, six male flies were dehydrated slightly by 5 minutes anesthetization with dry CO_2_ gas to promote prompt and synchronous drinking behavior upon awakening. The flies were then placed in a glass culture vial containing media adjusted to 160 ug/ml concentration RU486, and video tracking was initiated immediately when the flies awoke and righted themselves. Data were collected as 48 one-hour videos from each camera, analyzed by FluoreScore, and the average green fluorescence value for all pixels is plotted per minute, in light green (Figure 6a); in addition a smoothened plot was generated using local polynomial regression fitting (LOESS) and is indicated in dark green. Binning the pixels based upon intensity allows the data to be analyzed and visualized in the absence of background. The data were smoothed and plotted for the higher-intensity pixels (G3 and G4 bins; Figure 6b) and for the lower-intensity pixels (G1 and G2 bins; Figure 6c). Induction of stable GFP expression was first detected at 8 hours in the lower intensity range (G2) and at 12 hours in the higher intensity range (G3), and continued to increase through the end of the 48-hour tracking period. In the absence of drug no signal was detected in the G2–G4 bins (Figure 6d-f), because signal for these no-drug flies was limited to a low and relatively constant background contained entirely in the G1 bin (Figure 6f).

In the second experimental design the driver line Elav-GS was used, where the *Elav* gene promoter drives expression of the Gene-Switch transcription factor and the UAS-eGFP reporter specifically in the adult central nervous system [16], [17]. Induction of stable eGFP expression was first detected at 10 hours in the lower intensity range (G2; Figure 6i), and at 13 hours in the higher intensity range (G3; Figure 6 h), and continued to increase through the end of the 48-hour tracking period.

## Discussion

Several groups have reported methods for tracking 2D and 3D movement of Drosophila and other flies [7], [20]–[28]. Combining 3D tracking of Drosophila with simultaneous quantification of fluorescence allows for correlations between transgene expression, behavior, and aging [6], [8], [9]; however, application of this approach has been impeded by the difficulty of operating the systems. FluoreScore allows fluorescence to be quantified in groups of free-moving flies, and provides 3D movement patterns with simultaneous fluorescence quantification for single flies. The two-camera configuration, straightforward interface, and freely available software should make the FluoreScore system readily accessible to interested researchers. We note the system is readily adapted to tracking movement and quantifying fluorescent reporter molecules in *C. elegans* (data not shown).

Dividing the GFP data into bins of increasing pixel intensity (G1-G4) facilitates the analysis of the data. In the experiments presented here the background pixels, including those resulting from the low-level auto-fluorescence of Drosophila tissues, fell almost exclusively into the G1 bin, thereby facilitating their removal from subsequent data analyses. The signal in the G1 bin was sometimes slightly elevated in the first 1–2 hours of analysis (Figure 6c,f,i), and we conclude that this initial elevation of the background is most likely due to some residual food in the guts of the flies or fluorescent dust or fibers adhering to the flies during transfer.

One advantage of FluoreScore is that gene expression can be assayed longitudinally, such that the same animals are assayed at multiple time points or continuously. Longitudinal tracking of the induction of the UAS-eGFP transgene by Gene-Switch and RU486 feeding in adult flies demonstrated that eGFP fluorescence could be detected within 8 hours for tissue-general expression, and within 10 hours for CNS expression. Numerous steps are required for the induction of fluorescence, including animal feeding behavior, absorption of drug across the intestinal epithelium, and transport of the drug through the haemolymph to various internal tissues including the CNS. Once internalized, the drug must be taken up by cells, activate Gene-Switch conformation change and DNA binding, and Gene-Switch must in turn activate transcription and RNA synthesis; subsequently the newly-synthesized RNA must be processed, transported to the cytoplasm and translated. Finally, the new eGFP protein must undergo a multi-step, autocatalytic rearrangement to yield an active chromophore, and the maturation time for eGFP chromophore is reported to be ∼1.5 hours [29]. Previous studies of the time course of Gene-Switch activation employed cross-sectional assays of a group of animals, and required sacrificing a sub-set of the animals for assay at each time point. By staining sectioned head tissue for beta-galactosidase activity, Roman et al detected induction of a UAS-lacZ reporter in CNS within 3 hours after initiation of RU486 feeding to adult flies, with maximum levels achieved between 24 to 48 hours [15]. Similarly, Osterwalder et al detected GFP in animal extracts using Western blot assay within 5 hours after administration of drug to third instar larvae containing the Elav-GS driver and a UAS-GFP reporter [13]. The somewhat longer time observed here before eGFP fluorescence could be detected in live flies most likely includes the time required for eGFP chromophore maturation as well as the accumulation of sufficient eGFP fluorescence to be detected in the G2 bin by FluoreScore.

In conclusion, FluoreScore is freely available and user-friendly software that enables quantification of movement, behavior, and conditional gene expression in internal tissues in free-moving flies. FluoreScore should facilitate the in vivo assay of fluorescent molecules in a variety of future studies involving flies and other animals, including assays of gene expression, and potentially the assay of ROS-sensitive and metabolite-sensitive reporter molecules, FRET, and endogenous fluorescent compounds.

## Methods

### Drosophila Culture, Drug Treatments and Microscopy

Flies were cultured as previously described [16]. The Actin-GS[255B] and Elav-GS lines [16], [17], 3×P3-GFP[M1] line [9], [11], and multi-copy UAS-eGFP line “UAS-ultraGFP” [17], [19] have been previously described and characterized. Newly eclosed flies were collected over a period of 48 hours, maintained at 25°C at ∼10/vial in culture vials with food, and transferred to fresh food every other day until 7–10 days of age. Prior to drug feeding experiments, flies were placed on 5% sucrose solution overnight to clear food from the gut, as the food in gut has auto-fluorescence that can sometimes cause background signal. Immediately before beginning the experiments the flies were placed on the CO_2_ pad for 5 minutes without humidification of the gas: this dehydrates the flies slightly so that they will drink promptly upon awakening, thereby producing a more synchronous initiation of the experiment. The flies were then placed in vials containing 900 ul of grape agar media (Genesee Scientific) adjusted to 160 ug/ml RU486 (Mifepristone, Sigma). Grape agar media was found to produce no background fluorescence. Tissue-general and nervous system-specific induction of GFP in the flies was confirmed at the end of the tracking experiments using the Leica MZFLIII fluorescence stereomicroscope (data not shown).

### Video Assay Observation Chamber

Video assays were conducted with flies in a standard glass Drosophila culture vial, with the bottom of the vial and the vial stopper covered with non-reflective black cotton cloth. Plastic (polystyrene) Drosophila culture vials can also be used, but produce slightly more background that can be removed using the mask. Drug treatments were conducted in glass vials containing 900 ul grape agar media and the black cloth vial stopper.

### Silhouette Detection

Background subtraction was used to detect moving objects from the static camera views. A running Gaussian average was employed, that allows the background to be modeled independently at each 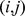 pixel. This model fits a Gaussian distribution to the intensity values of the pixel in the last 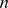 frames. Let 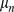 be the mean of the Gaussian model calculated after processing 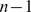 frames. A pixel is categorized as foreground if 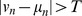, where 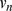 is the intensity value of the pixel and 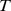 is a threshold value that can be determined empirically. Let 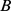 be a binary value that is 1 if the pixel is background and 0 otherwise. After classifying pixels in the current frame, 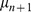
is calculated as shown below:
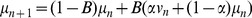
(1)


In this equation, 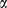 is known as the adaptation rate. As can be seen from Eq. 1, changing 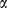
from 0 to 1 provides a compromise between stability and speed of update.

### 3D Reconstruction

Calibration of the two stationary cameras using the Camera Calibration Tools and CalibHelp software produces a camera projection matrix; detailed steps are provided in supplementary materials, experimental protocol (Protocol S1), and are also available for download from (http://fluorescore.cmb.usc.edu/, http://towerlab.usc.edu/). The camera projection matrix (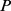) describes the mapping between the real 3D point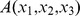 to the 2D point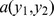 in the image:
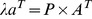
(2)where 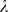 is a scaling factor. More specifically, using homogeneous coordination the relationship between the 2D point and the 3D point is as below:



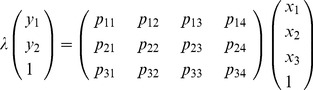
(3)The projection matrix models intrinsic parameters (focal length, pixel size, lens characteristics) and extrinsic parameters (rotation and translation) of the camera. Using the projection matrix the optical center (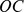) of the camera can be identified (Figure 3). Using the projection matrix for camera 1, the 3D line 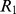 that has 2D projection point of 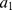
can be found. Performing the same procedure for camera 2 produces the other corresponding 3D line (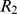). The intersection of the two 3D rays 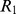
and 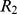
is the actual 3D position of the fly [30]. In practice, 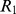
and 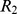 may not intersect due to inaccuracy in calibration and/or 2D measurements. We find the shortest line between 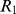
and 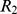 and consider the middle point of that line as their intersection (http://paulbourke.net/geometry/lineline3d/).

### Recording Images

The data storage capacity of the operating system employed here requires that files be smaller than 2GB; therefore Videograbber will record videos in separate files of a length of 108,000 frames (equal to 1 hr at 30fps). To facilitate processing of the multiple videos resulting from assays of multiple hours or days, a command line version of the FluoreScore software suite was created, and a batch file was generated to run each software module from the command line. To use the command line version, the GUI version of FluoreScore is used on the first video to determine processing parameters and create the mask, and this information is then used by the command line version as input arguments to analyze the remaining group of videos.

### Availability

All modules of the FluoreScore suite as well as future updates of the experimental protocol (Protocol S1) are freely available for download from our websites (http://fluorescore.cmb.usc.edu/, http://towerlab.usc.edu/).

## Supporting Information

Protocol S1 **Experimental Protocol.**
(DOCX)
