# Supplementary material for: Using GFP Video to Track 3D Movement and Conditional Gene Expression in Free-Moving Flies
Source: PLoS One. 2012 Jul 19;7(7):e40506. doi: 10.1371/journal.pone.0040506 (PMC3400653; doi:10.1371/journal.pone.0040506)
Supplement: Protocol S1 — Experimental Protocol. (DOCX) [file pone.0040506.s001.docx]

Supplementary Material: Using GFP video to track 3D movement and conditional gene expression in free-moving flies

Reza Ardekani, Yichuan Michelle Huang, Prathamesh Sancheti, Ramunas Stanciauskas, Simon Tavaré, John Tower.

# Experimental protocol

# Hardware requirements:

1. Desktop computer with FW800 PCI-E cards to connect the cameras.
2. Two video cameras (Grasshopper type GRAS-20S4C, Point Grey Research, Scottsdale, AZ)
3. 2 x 8mm Megapixel fixed focal lens (Edmund Optics Inc., Barrington, NJ)
4. Two blue LED lights (Blue 3 Watt LED MR16 bulb, Super Bright LEDs, Inc., St. Louis, MO)
5. MDF-GFP filter set (Thorlabs Inc, Newton, NJ)
6. Two MF469-35 filters (pass range: ~ 450nm-490nm)
7. Two MF525-39 filters (pass range: ~ 500nm-550nm)
8. Standard glass Drosophila culture vial as observation chamber
9. Aluminum Breadboard, 24" x 24" x 1/2" (MB2424, Thorlabs Inc., Newton, NJ)
10. 2 x Mounting Post, Length=14" (P14, Thorlabs Inc., Newton, NJ)
11. 2 x 1.5" Mounting Post Bracket(C1505, Thorlabs Inc., Newton, NJ)
12. 2 x 3" One-Arm Package, Two Knuckles, 3" Shaft (NT53-885, Edmond Optics Inc. , Barrington, NJ)
13. 2 x Mounting Post Base Ø2.40" X 0.50" High (PB2, Thorlabs Inc., Newton, NJ)
14. 2 x Dovetail Optical Rail, 3" (RLA0300 , Thorlabs Inc., Newton, NJ)
15. 2 x Extended Rail Carrier, 1" x 2", #8-32 Tapped Holes, 1/4" Counterbore (RC1, Thorlabs Inc., Newton, NJ)
16. Cardboard box

# Software requirements:

1. Flycap SDK (provided by Point grey research along with cameras)
2. Camera Calibration Tool, freely available for download at <http://www.cs.ucl.ac.uk/staff/Dan.Stoyanov/calib/>.
3. VideoGrabber (freely available at [http://code.google.com/p/video-grabber/](http://code.google.com/p/video-grabber/))
4. FluoreScoreSuite which has following modules:
   1. FluoreScoreGUI (version 1.00)
   2. FluoreScoreCMD (version 1.00)
   3. FluoreScoreSQ (version 1.00)
   4. FluoreScore3D (version 1.00)
   5. CalibHelp (version 1.00)
   6. fsAnalyzer (version 1.00)

User manual

# 1. Video recording

1. Create a new folder, e.g. under H:\GFP\ (e.g. name it DATE “4-9-11”).
2. Copy “VideoGrabber.exe” to the folder created in step 1.
3. Double click VideoGrabber.exe to open it. A Command line window will open. Enter inputs as mentioned below:

| 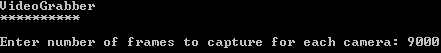 | Input “9000” for 5min; “7200” for 4min, etc (30 fps) |
| --- | --- |
| 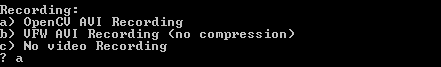 | We want to record videos using openCV, hence hit ‘a’ |
| 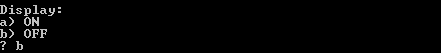 | One can choose ‘a’ to open display windows. But here we choose ‘b’ |
| Get prompt of # of cameras detected: 2  Number of cameras to use? | Enter “2”, hit return |
| 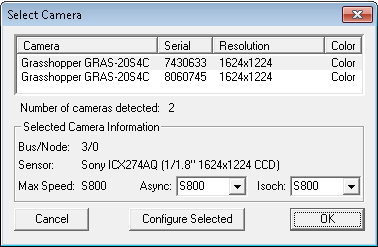 | Then the FlyCap window will pop up. Set configuration for both cameras at this time. Make sure that they have 800x600 resolution  YUV 422  and 30 Htz  (30 fps)  Click OK |
| 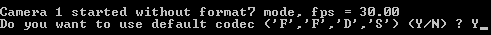 | Choose default Codec by entering “Y” |

1. Recording starts, once it is over a ‘Done’ message will appear. You must copy files to another folder or change file names if you want to record videos in the same folder. Otherwise if open VideoGrabber from the same folder old videos will be over-written!

# 2. Camera Calibration

1. Create a checkerboard as below, which contains NxN blocks with known dimension (e.g. 5mmx5mm) and mark each corner. (we chose N = 10)
2. Take five pictures using each camera. In each picture, let the checkerboard be inclined with different direction/angel as shown below:

| 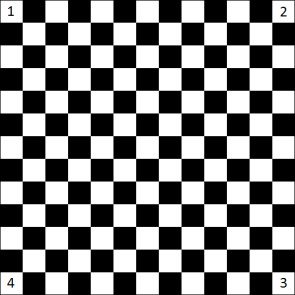 | 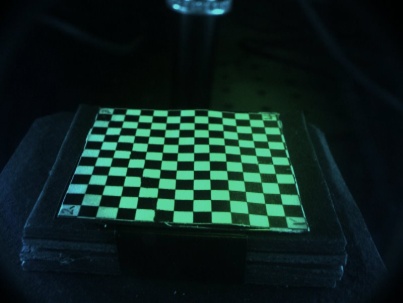 | 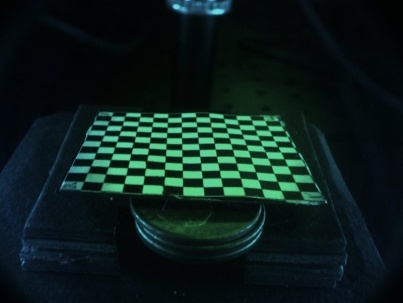 |
| --- | --- | --- |
| Checkerboard | position 1 | position 2 |
| 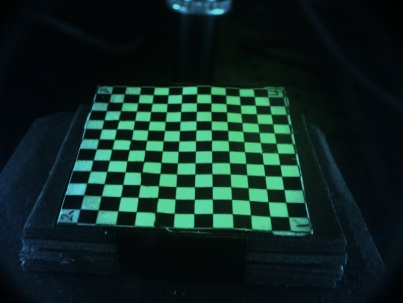 | 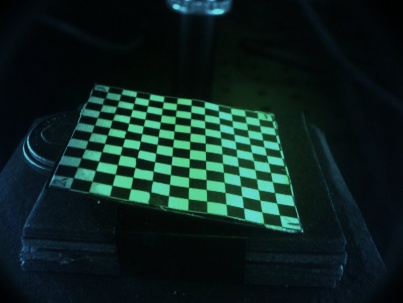 | 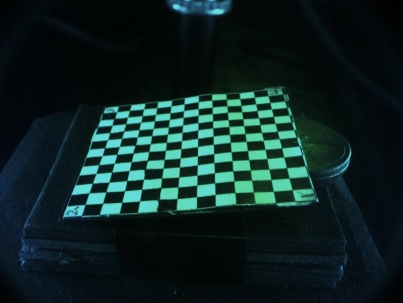 |
| position 3 | position 4 | position 5 |

1. Import all images into Calibration tool and calibrate each camera as described in following table.
2. Copy “HelpCalib.exe” into the folder that contains result of calibration. Double click on HelpCalib.exe to run the program. A file named “projMat.txt” will be created in the same folder. Close the program by pressing any key. *“projMat.txt”* is exactly what we need in FluoreScore3D.exe

| STEP 1: Click on the logo as left twice in order to create two different cameras. | 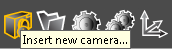 |
| --- | --- |
| STEP 2: Import images into each of camera. Please import them one by one and in the same order for both cameras. | 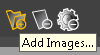 |
| STEP 3: Double click on first image and set parameters for the camera. | 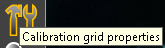  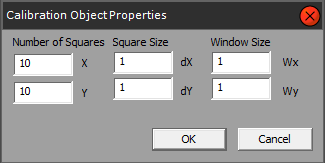 |
| STEP 4: Select grid of image. Click on image from corner1-> corner 2-> corner 3-> corner 4. Make sure that the order is same for all images. Calibration Tool will extract the points of intersection for grids. Make sure these points are not off from intersections. | 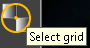  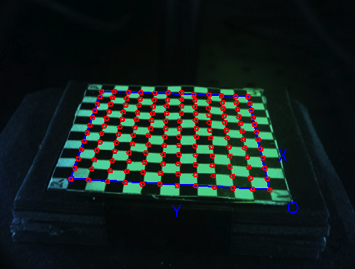 |
| STEP 5: Do STEP 3 for all images. |  |
| STEP 6: Select camera 1. Click on Calibrate button. It will generate intrinsic and extrinsic parameters of the camera. Do the same operation for camera 2. | 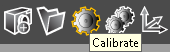 |
| STEP 7: Click on SAVE icon to save project. . Once the project is saved, multiple files will be created which include intrinsic/extrinsic parameters for each camera. | 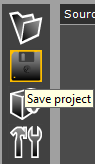 |
| **Camera Calibration steps** | |

# 3. Finding processing parameters

**Goal:** Indicating threshold, masking area and ROI for one experiment.

| 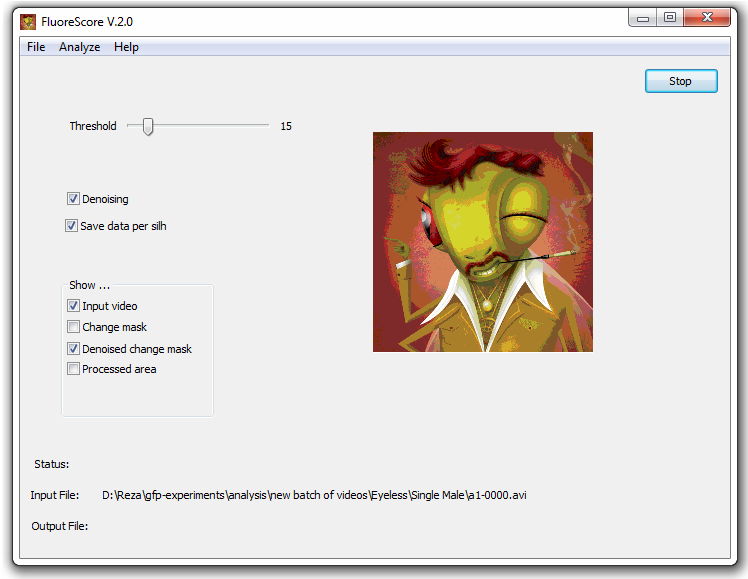 |
| --- |
| FluoreScoreGUI |

1. Open FluorescoreGUI and under the **file** menu click on ‘**open…**’ and indicate the input video.
2. To set threshold use slider. If the output filename has not been indicated it will ask for an output file name. Enter that and continue. Threshold can be set between 0 and 127. The larger the threshold is, the less noise there will be. To find an appropriate threshold you should see the output video. Make sure that under show box input video and processed area are checked. Adjust threshold such that flies are detected (blue color) in the majority of frames, and background (scattered) pixels are eliminated. Record the value used. Use this value across any additional samples so data is analyzed consistently.
3. To set the processing mask open the input video and under the **analyze** menu choose **mask**. Draw free hand closed loop around areas to be excluded, and then close. Use this option to exclude regions of glare or other background from the analysis. After you have closed all the masking windows, a jpg file with name *videofilename_mask.jpg* will appear in the folder that contains the original video files.
4. To select region of interest (ROI) choose **Custom ROI** under the **Analyze** menu. A window with a frame of video will pop up. Right -click on image to select the upper left point; and don’t release right-click until you reach the lower right point of the rectangular ROI. You can do this as many times as you want until you get your desired ROI. Once you are done close the frame window. A text file called *videofilename _roiPoints.txt* will be created in the same folder that contains the video file.
5. Repeat the same process for the second video.

# 3. Processing videos

In this section, we describe the command line options for FluoreScoreCMD, FluoreScoreSQ and FluoreScore3D.

## 3.1) FluorScoreCMD

| Command line options: |
| --- |
| FluoreScoreCMD.exe <first view video file(.avi)> <threshold for first view (between 0 and 127) > <second view video file(.avi)> <threshold for second view (between 0 and 127) > <Display (*y for yes;n for no*)> <Number of frames to be processed (*-1 to process the whole video)>* <mask Image for view1(.jpg)> <ROIleftUpperX> <ROILeftUpperY> <ROIrightDownX> <ROIrightDownY> <mask Image for view2(.jpg)> <ROIleftUpperX> <ROILeftUpperY> <ROIrightDownX> <ROIrightDownY> |
| Example: |
| FluoreScoreCMD.exe AviFilecam0.avi 15 AviFilecam1.avi 15 n -1 AviFilecam0_mask.jpg 160 80 570 560 AviFilecam1_mask.jpg 230 100 550 530 |

## 3.2) FluoreScoreSQ

| Command line options: |
| --- |
| FluoreScoreSQ.exe <first view info file (.csv)> <second view info file (.csv)> <combined output (.csv) <flyNumber(*should be 1*)> <threshold for the size of flies (in pixel)> |
| Example: |
| FluoreScoreSQ.exe view0.csv view1.csv finalOutput.csv 1 20 |

For threshold for size of the fly we use 15-20 for eyeless-GFP (eyes), 20 for esg-GFP(gut) and 50 for hsp70GFP (whole body).

## 3.3) FluoreScore3D

| Command line options: |
| --- |
| FluoreScore3D.exe <2D position info file from view 1> <2D position info file from view 2> <projection matrices file (projmat.txt)> <output 3D positions (.csv)> |
| Example: |
| FluoreScore3D.exe view1_pos.csv view2_pos.csv projmat.txt 0_3dpos.csv |

Make sure *projmat.txt* is in the same folder with videos or give the FluoreScore3D.exe the full path to projMat.txt. For example “*D:\\GFP\\Experiment1\\projMat.txt*”.

## 3.4 Batch processing

### 3.4.1) Short experiments

If the experiments are less than one hour, we will have one video file for each view. After finding parameters using FluoreScoreGUI, processing videos FluoreScoreCMD, FluoreScoreSQ and FluoreScore3D can be called in a .BAT file to facilitate processing. To do so create a new file with Notepad and copy paste the commands in the table below and save the file with ‘.bat’ extension. Make sure you change the processing parameters and filenames according to your experiment. Moreover when there is only one fly, we not only get GFP data but also 3Dposition of the fly over the time. In this case we call FluoreScore3D to reconstruct 3D position of the fly.

| # for number of flies = 1  @echo on  FluoreScoreCMD.exe AviFilecam0.avi 15 AviFilecam1.avi 15 n -1 AviFilecam0_mask.jpg 160 80 570 560 AviFilecam1_mask.jpg 230 100 550 530  FluoreScoreSQ.exe view0.csv view1.csv finalOutput.csv 1 20  FluoreScore3D.exe view0_pos.csv view1_pos.csv projmat.txt 3dpos.csv |
| --- |
| # for Number of flies = **N** (N<>1)  @echo on  FluoreScoreCMD.exe AviFilecam0.avi 15 AviFilecam1.avi 15 n -1 AviFilecam0_mask.jpg 160 80 570 560 AviFilecam1_mask.jpg 230 100 550 530  FluoreScoreSQ.exe view0.csv view1.csv finalOutput.csv view0_pos.csv view1_pos.csv 1 20 |

### 3.4.2) Long experiments

To facilitate processing experiments that are long (e.g. 24 hours experiments) user can use the bat file that is provided in FSAnalyzer called “FluoreScoreBatch.bat”. The usage of that is as below. You should define your processing parameters (e.g. mask image and ROI) in the file.

| Command line options: |
| --- |
| FluoreScoreBatch.bat <first hour> <last hour> |
| Example: |
| FluoreScoreBatch.bat 1 24 |

# 4) Data Analysis (FSAnalyzer)

## 4.1) Short Experiments

We do some basic statistical analysis and plotting using R. Open *fsAnalyzer_shortExperiments_main.r*. The parameters that need to be set (from setwd to nameofbin ) are listed below:

| **Parameter** | **Function** |
| --- | --- |
| **setwd("")** | Set working directory. Put the folder directory, which contains all the **.CSV** files, within double quotations.  e.g. setwd("H:/GFP/100924") |
| **caseFileName** | Name of case.CSV file. (No need to put .CSV)  e.g. caseFileName = "mycasefile" ata |
| **ctrlFileName** | Name of ctrl.CSV file. (No need to put .CSV)  e.g. ctrlFileName = "myctrlfile" |
| **experimentName** | Name of experiment, which will be shown as a title for the figures.  e.g. experimentName = "100727 GFP VS non-GFP" |
| **doCompare** | If there are a case and control, input ”TRUE” otherwise input “FALSE”. |
| **fileNumber** | Number of input GFP files. (1 or multiple) |
| **flyNumber** | Number of flies. |
| **videoLength** | Length of input videos (in frame, e.g. 9000 or 108000) |
| **smoothingWindowSize** | Window Size for smoothing (frame base).  e.g smoothingWindowSize = 1801 for about 1 minute window. 4 minutes = 7201. Note: the number should be odd. |
| **binName** | Name of the bin that you want to plot. If multiple bin information is needed, one can simply input any bin name separated by “+”.  e.g nameofbin = "g3Num" or “g4NUM”. nameofbin = "g3Num+g4Num+gAvg+g4Sum+r1Avg+b4Sum”  *one can use any combination of any bin(g/r/b, 1/2/3/4, Sum/Num/Avg)! |
| **CombinedSum/**  **CombinedFlag** | Name of the bin that you want to add. As you are going to  Add 2 or more “+” sign will be there.  e.g CombinedSum = “g3Sum+g4Sum”  If you do not want to add bin then make CombinedFlag = “FALSE” otherwise “TRUE”. |
| **DummyFileNumber/ StartDummy** | If you want to process multiple files simultaneously you can enter start file number and end file number. For example StartDummy = 1 and DummyFileNumber = 15. Program will process all the files from 1 to 15 |

To choosing Bin (G4-G1), start with the highest bin that has pixels for any sample. If that bin does not contain pixels for each sample, add the next highest bin and so on.

Here is an example of input parameters:

| setwd("H:/GFP/4-11-11/C7-2_FvsM/paper");  caseFileName <- "C7-2_F_3";  ctrlFileName <- "C7-2_M_3";  experimentName <- "4-11-11_C7-2"; # name of experiment  doCompare <- TRUE;  fileNumber <- 1;  flyNumber <- 2;  videoLength <- 7200; # length of video (in frame)  smoothingWindowSize <- 900; #window size for Smoothing  binName <- "g3Sum+g4Sum"; # name of the bin that you are interested, i.e g4num  CombinedSum <- "g3Sum+g4Sum";  CombinedFlag <- TRUE; |
| --- |

## 4.2) Long Experiments

In long experiments (i.e. 24 or 48 hours) we are mostly interested in measuring the change of GFP signal in time. To do so, open ‘*fsAnalyzer_longExperiments_main.r*

| **Parameter** | **Function** |
| --- | --- |
| **experiment_name** | This will be used to name the figures |
| **Filename_Prefix** | csv filenames should be in a specific format |
| **PathToCSVFiles** | Path to the folder that contains .csv files generated by FluoreScoreSuite(Usually the same folder that contains videos |
| **firstHour** | First hour to process |
| **LastHour** | Last hour to process |

*It will generate a .tif in the same folder of csv files that contains plot of average, G1, G3, and G4 for the course of time.*

Here is an example of input parameters:

| experiment_name ="255B";  filename_Prefix = "Fly_"; # csv here the filenames are Fly_1.csv, Fly_2.csv, ..  pathToCSVFiles = "G:/newFluoreScoreTest/5-12-11/";  firstHour = 1; # first hour to process  lastHour = 24; # last hour to process |
| --- |

# 5. Vial setup

1. Adjust luminance / focus of both cameras. Try to make the setting as close as possible.
2. Adjust distance between platform and lights. For 24 hours/48 hours videos, make sure to put back both lights and make the distance between light and vial to be 15cm.
3. When during experiment, try to put vial in exactly the same place. (There is a pattern of circle on platform)


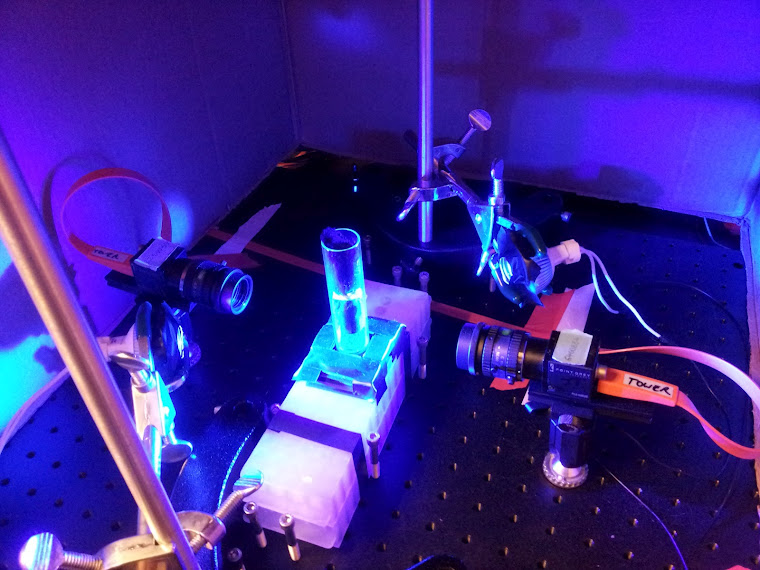


Camera 1

Camera 2

Light 1

Light 2

# 6) Supplementary package

This package contains three folders:

**1- PloSONE_ExperimentData:**

This folder contains data files (.csv) for three experiments (255B with drug, Elav-GS with drug and 255B without drug). ‘toPlot.r’ generates figure 6 in the paper.

**2- Running Sample**

This folder contains:

* Input files:

- View a AVI file (eyeless_a1800.avi)

- View b AVI file (eyeless_b1800.avi)

- Projection matrices for 3D reconstruction (projMat)

* Intermediate output files:

- eyeless_a1800.csv

- eyeless_b1800.csv

- pos2d_a.csv

- pos2d_b.csv

* Final output files:

- GFP signal for the experiment (combinedGFPOutput.csv)

- 3D position of fly (pos3D.csv)

Rename run.txt to run.bat and run it, it will generate final (and intermediate) output files from input files. Use plot3D.r to see the 3D path (Figure 4 in the paper).

**3- Sample footage:**

This folder contains two footages, one for single fly and the other one for multiple flies. (FluoreScoreSuite_Multiple_Flies.avi and FluoreScoreSuite_SingleFly.avi.). These videos show how the processing is done.
